# Angioplasty in acute middle cerebral artery stroke due to atrial fibrillation selected by CT perfusion: a case report

**DOI:** 10.1186/1865-1380-4-23

**Published:** 2011-06-06

**Authors:** Andrea Saletti, Ilaria Morghen, Luca Finessi, Enrico Fainardi

**Affiliations:** 1 Neuroradiology Service, Department of Neuroscience, S.Anna University Hospital, C.so Giovecca 203, 44100 Ferrara, Italy; 2 Anaesthesiology and Critical Care Department, S.Anna University Hospital, C.so Giovecca 203, 44100 Ferrara, Italy; 3 Neuroradiology Service, Department of Neuroscience, S.Anna University Hospital, C.so Giovecca 203, 44100 Ferrara, Italy

## Abstract

We report the experience of a case of acute stroke in a patient affected by Rendu Osler syndrome and atrial fibrillation. The combination of dynamic computerized tomography perfusion scans and the use of a high-compliance balloon allowed increasing the treatment window for intra-arterial recanalization over 6 h after stroke onset in a patient with middle cerebral artery occlusion.

## Background

Stroke is the third leading cause of death in the United States, Canada, Europe, and Japan.

Currently, thrombolysis of the clot responsible for an ischemic event is proving to be an effective therapy for improving the patients' condition following an acute stroke.

One of the most important elements in obtaining clinical success is how early treatment is first established: early treatment within 3 h for intravenous recombinant tissue plasminogen activator (rt PA) and within 6 h for IA (intra-arterial) thrombolysis shows significant post-clinical improvement after 90 days and reduced incidence of cerebral hemorrhage.

Improved imaging, distinguishing ischemic penumbra from irreversibly infarcted tissue, could be a criterion more accurate than the duration of symptoms for selecting patients to undergo therapy [1].

Recent advances in the field of neurointerventional radiology, with the development of different clot retrievers, and high-compliance balloons and stents, have made IA recanalization feasible and afford safe access to the major intracranial blood vessels by a percutaneous transfemoral approach under local anesthesia.

Successful recanalization is associated with improved outcome after acute ischemic stroke [2]. Treatment with mechanical thromboembolectomy techniques is proposed for failed recanalization after thrombolysis or for patients with contraindications for thrombolytic therapy. However, mechanical recanalization techniques are not always successful.

In a MERCI trial, the association of mechanical recanalization and IA therapy showed an overall recanalization rate of 68% [3].

Currently, there is an increasing tendency to treat acute stroke in a multimodal way, including angioplasty with or without stenting [2-6].

In this case report we show angiographic recanalization and clinical improvement in a patient on anticoagulant therapy who had suffered an acute MCA (middle cerebral artery) stroke because of atrial fibrillation and who benefitted from angioplasty.

The purpose of this paper is to document a case of acute stroke in a patient with middle cerebral artery occlusion using a combination of dynamic CT (computerized tomography) perfusion scans to perform tissue perfusion and high-compliance balloon techniques, with a treatment window for intra-arterial thrombolysis longer than 6 h from the stroke onset.

## Case presentation

A 70-year-old right-handed female was brought to the emergency room 35 min after the sudden onset of a stroke. She arrived at the hospital at 11.35 a.m. with dysarthria, right homonymous hemianopsia, dense right hemiplegia, and partial gaze palsy.

Neurological deficit on presentation was assessed using the National Institutes of Health Stroke Scale (NIHSS) with a score of 24. She had a past medical history of hypertension, hyperlipidemia, atrial fibrillation, and hereditary hemorrhagic telangiectasia.

On general medical evaluation she was alert but disorientated. Her blood pressure was 170/80, serum glucose value 89 mg/dl, and cholesterol 213 mg/dl.

**Figure 1:**
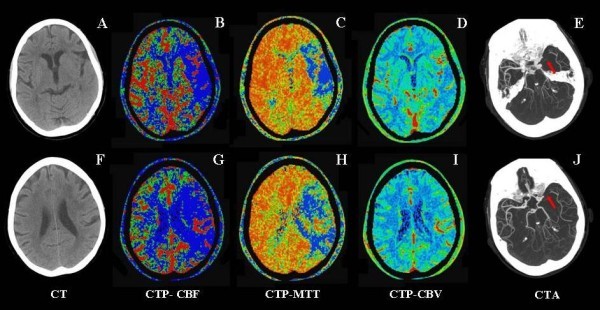
**CT protocol performed 3 h after stroke onset**. *A and F*: unenhanced CT scans (ASPECTS levels) show the presence of an area of slight hypoattenuation in the left insular ribbon and in the ipsilateral frontal cortex (ASPECTS = 8); *B-D and F-H*: CTP maps (ASPECTS levels) show the occurrence of a large mismatch CBF > CBV or MTT > CBV in the territory of left middle cerebral artery; *E and J*: axial CTA MIP images document the occlusion of M1 segment of left middle cerebral artery (*arrows*).

At 12:00 she underwent an emergent brain CT scan, which revealed no hemorrhage and only a mild front left hypodensity in the insular area. The Alberta Stroke Program Early CT Score was 8. CT angiogram showed an occlusion of the left M1 trunk, and CT perfusion (CTP) revealed a mismatch among the CBF (cerebral blood flow), MTT (mean transit time), and CBV (cerebral blood volume) maps (Figure 1).

All stroke patients arriving at our hospital undergo an emergent brain CT scan, CT angiogram (CTA), and CT perfusion (CTP), according to a protocol agreed upon by neurologists and intensivists.

On the basis of the presence of penumbra, the decision was taken to transfer the patient to an angiographic ward.

Because the angiographic suite was busy with other procedures, an angiography was performed 6 h after the onset of symptoms without any change in her clinical condition. It confirmed an occlusion of the main M1 trunk with excellent collateral circulation from the leptomeningeal anterior cerebral artery and retrograde opacification of the distal M1 segment.

An angiogram was obtained by pulling back the microcatheter to show the extent of the thrombus.

According to exclusion criteria of the SITS-MOST Study Protocol [7], Rendu Osler syndrome represents an absolute contraindication for rt PA infusion.

We opted for a mechanical thrombectomy performed more than 6 h after the onset of the stroke with a Hyperform 4 mm × 7 mm balloon angioplasty (EV3) positioned inside the thrombus. Recanalization was achieved with rapid inflations (duration of the inflation, maximum 10 s) at different levels of the M1 trunk.

**Figure 2:**
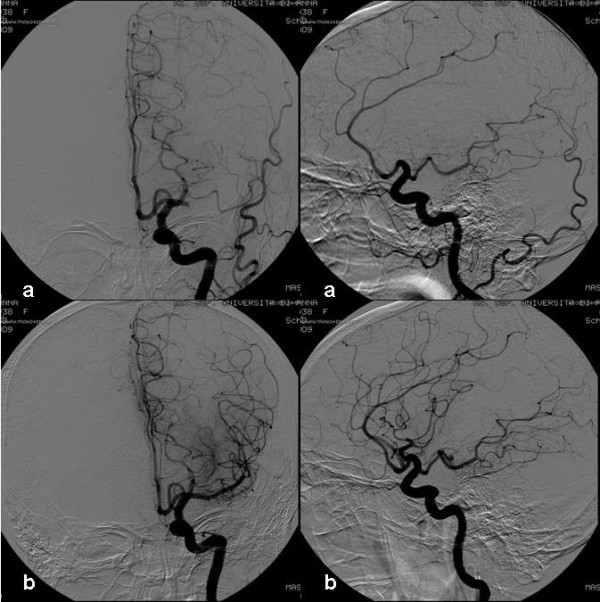
**Left internal carotid angiogram shows occlusion of M1: control angiogram after a couple of gentle inflations of a hyperform balloon and recanalization of the middle cerebral artery**.

At the end of the procedure, the artery was recanalized. Thrombolysis in Cerebral Infarction Perfusion Categories (TICI) was grade 3 (Figure 2).

**Figure 3:**
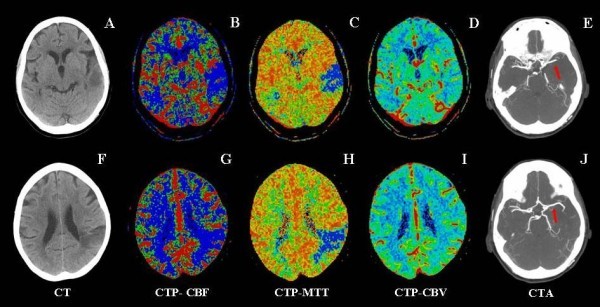
**CT protocol performed at 7 days after stroke onset**. *A and F*: unenhanced CT scans (ASPECTS levels) show the appearance of two areas of hypoattenuation in the left temporal and parietal cortex; *B-D and F-H*: CTP maps (ASPECTS levels) demonstrate the persistence of two areas of hypoperfusion in CBF and MTT maps and show the normalization of the CBV defect in the territory of the left middle cerebral artery; *E and J*: axial CTA MIP images shows the complete recanalization of the M1 segment of the left middle cerebral artery (*arrows*).

Follow-up CTP after 24 h and 7 days revealed persistence of the mismatch (Figure 3).

NIHSS score at 24 h was 8, and the Rankin scale at 3 months documented a slight disability (Modified Rankin Score 2).

## Discussion

This case shows that angioplasty of an acute embolic occlusion of the MCA may represent the correct course of action to rapidly restore cerebral flow, especially when the thrombus extends beyond the lenticulostriate arteries and in patients with contraindications for thrombolysis or who are on anticoagulant therapy, since drug reduction would be detrimental to the patient's health.

Many studies have shown the feasibility of endovascular recanalization (PTA) in cases of acute stroke [8,9].

Nakano et al. performed a retrospective comparison of 34 patients with acute MCA trunk occlusion who had been treated with direct PTA (followed by thrombolytic therapy in 21 patients) with 36 similar patients who had been treated with thrombolytic therapy alone [10].

Partial or complete recanalization was achieved in 91.2% of the first group as opposed to 63.9% of the second. Symptomatic intracerebral hemorrhage was observed in 2.9% versus 19.4%, and a good outcome (Modified Rankin Score ≤ 2) was reached in 73.5% versus 50% of the patients, respectively.

The majority of patients were treated with semicompliance balloons, typically coronary balloons, whereas Mangiafico et al. [11] and Lum et al. documented a series of 21 and 9 patients, respectively, treated with a combination of IA drugs and HyperGlide balloons.

The Multi MERCI trial, designed in part to test the performance of a new generation of thrombectomy devices, found an increased though not statistically significant rate of recanalization of intracranial vessels [3].

The potential risks associated with direct PTA include arterial rupture, spasm, and distal embolization [12-14].

In embolic occlusion, the balloon catheter should only penetrate the embolus, and dilatation force applied to the vessel wall is usually less than that used in the treatment of intracranial vascular stenosis [15].

Another potential drawback of this technique is the theoretical risk of occluding the lenticulostriate arteries because of the displacement of the clot. The use of thrombolytic agents, particularly local intra-arterial infusion of highly concentrated or high-dose thrombolytic agents into the ischemic tissue, may be the greatest risk factor in symptomatic hemorrhage.

For these reasons, mechanical clot removal without the use of thrombolytic agents may be the ideal treatment for acute ischemic stroke, especially in patients with a high hemorrhagic risk and increasing time from stroke onset, when the risk of hemorrhage increases.

In addition to clinical features, perfusion imaging techniques combined with MRI (magnetic resonance imaging) or CT are mandatory to recognize a clear demarcation of irreversible damaged infarcted and ischemic but still recoverable tissue, to accurately determine which patients may benefit from reperfusion, especially those with unknown time of stroke onset or who are outside the recommended 6-h time window for therapy [16-18].

The new MR techniques of diffusion-weighted and perfusion-weighted imaging, including in a diagnostic protocol of acute stoke patients, identified ischemic brain regions and detected impaired perfusion. CT is an essential diagnostic requirement in a stroke center to exclude patients with intracerebral hemorrhage and extensive demarcation of ischemic infarction.

In these instances, the presence of mismatch may justify mechanical revascularization in order to reduce potential hemorrhagic complications, as in our case.

## Conclusion

Salvage angioplasty of acutely occluded intracranial arteries with a high-compliance balloon may represent an option for a correct first course of action to achieve rapid recanalization, especially in MCA occlusion beyond the lenticulostriate arteries and in patients on anticoagulant therapy or having other contraindications for thrombolysis.

Perfusion imaging techniques are fundamental in assisting decision-making in cases of arterial occlusion.

## Abbreviations

IA: intra-arterial; CT: computerized tomography; MRI: magnetic resonance imaging; MCA: middle cerebral artery; NHSS: National Institutes of Health Stroke Scale; CTP: perfusion computerized tomography; CBF: cerebral blood flow; MTT: mean transit time; CBV: cerebral blood volume; rt PA: recombinant tissue plasminogen activator; TICI: Cerebral Infarction Perfusion Categories; PTA: endovascular recanalization; SITS-MOST: Safe Implementation of Treatment in Stroke-Monitor Observational Study.

## Competing interests

The authors declare that they have no competing interests.

## Authors' contributions

Preoperative evaluation, conception of the paper, literature search and manuscript draft carried out by AS. Contributions to conception and design were made by EF. Drafting and revising of the manuscript was performed by LF. Literature search, help in drafting and revising of the manuscript was provided by IM. All authors have read and approved the final manuscript. We had the paper proofread by BA. ESL Canadian and UK English teachers.

## Consent

Written consent for the publication of this case report and any accompanying images was obtained from the patient. A copy of the written consent is available for review from the Editor-in Chief of this journal.
